# Epistemic Benefits of Elaborated and Systematized Delusions in Schizophrenia

**DOI:** 10.1093/bjps/axv024

**Published:** 2015-07-15

**Authors:** Lisa Bortolotti

**Affiliations:** University of Birmingham Birmingham B15 2TT, UK, l.bortolotti@bham.ac.uk

## Abstract

In this article I ask whether elaborated and systematized delusions emerging in the context of schizophrenia have the potential for epistemic innocence. Cognitions are epistemically innocent if they have significant epistemic benefits that could not be attained otherwise. In particular, I propose that a cognition is epistemically innocent if it delivers some significant epistemic benefit to a given agent at a given time, and if alternative cognitions delivering the same epistemic benefit are unavailable to that agent at that time. Elaborated and systematized delusions in schizophrenia are typically false and exemplify failures of rationality and self-knowledge. Empirical studies suggest that they may have psychological benefits by relieving anxiety and enhancing meaningfulness. Moreover, these delusions have been considered as adaptive in virtue of the fact that they enable automated learning to resume after a significant disruption caused by incorrect prediction-error signalling. I argue that such psychological benefits and adaptive features also have positive epistemic consequences. More precisely, delusions can be a means to restoring epistemic functionality in agents who are overwhelmed by hypersalient experiences in the prodromal stage of psychosis. The analysis leads to a more complex view of the epistemic status of delusions than is found in the contemporary philosophical literature and has some implications for clinical practice.

**1** *Introduction*
**2** *Types of Delusions*
**3** *What Is Wrong with Elaborated and Systematized Delusions?*
**4** *Finding Life Meaningful*
**5** *Learning Resumed*
**6** *Epistemic Innocence*
**7** *Epistemic Benefit*
**8** *No Alternatives*
**9** *Conclusions and Implications*

## 1 Introduction

Delusions are regarded as a paradigmatic instance of irrationality and as the mark of madness. In this article I want to defend the apparently implausible thesis that some delusions have epistemic benefits.

In Section 2 I describe three types of delusions: (i) monothematic and circumscribed delusions emerging from brain damage or cognitive deterioration; (ii) monothematic delusions that seem to play a defensive function, and emerge from trauma or after adversities; and (iii) elaborated and systematized delusions in schizophrenia (hereafter, ESDs). In Section 3 I consider the epistemic costs associated with ESDs in terms of failures of irrationality and self-knowledge. In Sections 4 and 5 I turn to the potential benefits of ESDs in terms of relieving anxiety, enhancing meaningfulness, and allowing learning to resume after the disruption caused by incorrect prediction-error signalling. In Section 6 I introduce the notion of ‘epistemic innocence’ as the status of cognitions that have epistemic costs but also significant epistemic benefits. I describe two conditions for the epistemic innocence of delusions: ‘epistemic benefit’ and ‘no alternatives’. In Sections 7 and 8 I make a case for the view that ESDs have the potential for satisfying both conditions (with some qualifications). In Section 9 I consider the implications of the view for epistemic evaluation, the reconceptualization of delusions, and clinical interventions on people with ESDs.

## 2 Types of Delusions

Clinical delusions are symptoms of psychiatric disorders such as schizophrenia and dementia. An example is the delusion of persecution, the belief that one is being threatened by others and is going to be harmed. An agent may interpret clues in her environment to mean that others are being hostile to her and intend to harm her, even though there is no immediate threat. In the psychiatric literature, delusions are characterized as fixed beliefs with implausible content.^1^ They are defined on the basis of their surface features and are thought to exemplify failures of rationality and self-knowledge.

Here are some helpful definitions of delusions:

A false belief based on incorrect inference about external reality that is firmly held despite what almost everyone else believes and despite what constitutes incontrovertible and obvious proof or evidence to the contrary. The belief is not ordinarily accepted by other members of the person's culture or subculture (i.e. it is not an article of religious faith). When a false belief involves a value judgment, it is regarded as a delusion only when the judgment is so extreme as to defy credibility. (American Psychiatric Association [2013], p. 819)

A person is deluded when they have come to hold a particular belief with a degree of firmness that is both utterly unwarranted by the evidence at hand, and that jeopardises their day-to-day functioning. (McKay *et al.* [2005], p. 315)

Delusions are generally accepted to be beliefs which (a) are held with great conviction; (b) defy rational counter-argument; and (c) would be dismissed as false or bizarre by members of the same socio-cultural group. (Gilleen and David [2005], pp. 5–6)

The definitions above characterize delusions on the basis of their negative epistemic features, including lack of warrant, fixity, resistance to counterargument, and implausibility. Depending on the type of delusions, other features can be observed. So-called deficit delusions are typically monothematic (that is, involve just one theme) and are often circumscribed (that is, they do not interact with other beliefs). They can be the result of brain damage or cognitive deterioration. Examples include the Capgras delusion (the belief that a loved one has been replaced by an impostor) and mirrored-self misidentification (the belief that there is a stranger in the mirror when one looks at one’s own reflection). So-called motivated delusions are usually monothematic delusions that seemingly protect the person from low self-esteem or negative emotions. They are a response to trauma or previous adversities. Examples are the reverse Othello syndrome (the belief that one’s romantic partner is faithful when she is not) and anosognosia (the denial of illness, for instance, the paralysis of a limb). Some delusions in schizophrenia are systematized and elaborated (ESDs). They may involve several themes, and they can turn into complex, all-encompassing narratives. Examples include the delusion of grandeur (the exaggerated belief in one’s self-worth) and the delusion of reference (the belief that some events are highly significant).

This way of identifying types of delusions does not necessarily have implications for classification, diagnosis, or aetiology. The three types mainly track surface features of delusions and thus are useful in determining the relevant costs and benefits of delusions from a psychological and epistemic point of view. In the rest of the article I shall focus on delusions of the third type (ESDs), although I believe that some considerations also apply to motivated delusions (see Bortolotti [2015]).

## 3 What Is Wrong with Elaborated and Systematized Delusions?

In this section I will describe some of the characteristics of ESDs leading to irrationality, failures of self-knowledge, and impaired functioning. First, ESDs can be characterized as irrational beliefs, in that they are implausible, they do not respond to evidence, and they are not consistently reflected in behaviour. Although the presence of abnormal experiences may provide some justification for the initial acceptance of a delusional hypothesis (see Coltheart *et al.* [2010]), the delusional hypothesis is implausible given what the agent already knows (McKay [2012]). Delusions emerging in the context of schizophrenia are also very resistant to counter-evidence: agents discount evidence that speaks against the content of their delusion and provide confabulatory reasons to accommodate recalcitrant facts.

Second, delusions may be signalling a failure of self-knowledge. A person may be in two minds about the content of the delusion. Classical examples are the man with persecutory delusions who claims that nurses in the hospital want to poison him, but keeps eating the food they give him (Gallagher [2009]), and the woman who claims to be the queen, but does not behave like royalty (Bleuler [1924]). People with ESDs often have a distorted conception of their own physical and mental boundaries, and they may attribute their own movements or thoughts to others. They may claim that their neighbour is inserting thoughts into their head or that their arm is either controlled by someone else or not really theirs. False reports about past experiences and life events are common. That is why delusions have also been described as ‘unreliable autobiographies’ (Gerrans [2009]), where salience is attributed to irrelevant events and there is a lack of correspondence with reality.

Although I have focused so far on the epistemic costs of ESDs, such delusions are costly in other ways too. They typically impair good functioning via a negative impact on socialization and well-being. Delusions can be preoccupying and distressing, interfering with the agent’s social life. This extract from a first-person account of schizophrenia illustrates the point very well:

I increasingly heard voices (which I'd always called ‘loud thoughts’ or ‘impulses with words’) commanding me to take destructive action. I concluded that other people were putting these ‘loud thoughts’ in my head and controlling my behavior in an effort to ruin my life. I smelled blood and decaying matter where no blood or decaying matter could be found (for example, in the classrooms at school). I had difficulty concentrating, I fantasized excessively, and I had trouble sleeping and eating. (Bockes [1985], p. 488)

People usually come to the attention of healthcare professionals and are diagnosed with delusions when they do not sleep properly, experience social withdrawal, cannot keep their jobs or continue their studies, and cause concern to their families, employers, neighbours, or the police. Although (as we shall see) there are some cases of ‘successful psychotics’ (Hosty [1992]) whose lives do not seem to be as severely affected by their delusions, before we turn to the putative benefits of delusions we should remember that, for the great majority of sufferers, delusions seem to be a constant source of unhappiness.

## 4 Finding Life Meaningful

Considerations about the epistemic costs of ESDs and their adverse effects on functioning might have ruled out any investigation into their potential benefits, but psychologists have looked into the possibility that ESDs contribute to people finding their lives more meaningful and coherent. As a result of the studies I shall describe in this section, the relationship between delusions and well-being appears more complex that one might have expected.

In the prodromal stage of psychosis, agents are bombarded with stimuli presented to them as salient (Kapur [2003]). The agent does not know how to interpret the hypersalient stimuli and gets anxious. The world becomes difficult to understand and predict:

This general delusional atmosphere with all its vagueness of content must be unbearable. Patients obviously suffer terribly under it and to reach some definite idea at last is like being relieved of some enormous burden […] The achievement brings strength and comfort […] No dread is worse than that of danger unknown. (Jaspers [1963], p. 98)

Anomalous experiences create ‘puzzlement, anxiety, and a search for an explanation’ (Maher [2006]). The agent is constantly expecting something important to happen, until the delusional hypothesis is endorsed. This is the ‘a-ha moment’, the revelation, putting an end to the often long stage of anxious expectation that Klaus Conrad vividly describes in his work on schizophrenia (Mishara [2010]). When the delusion is formed, uncertainty is overcome and previously puzzling experiences are made sense of. In this context, delusion formation can be seen as adaptive.

Glenn Roberts argues that delusion formation allows agents to attribute meaning to experience:

Delusion formation can be seen as an adaptive process of attributing meaning to experience through which order and security are gained, the novel experience is incorporated within the patient's conceptual framework, and the occult potential of its unknownness is defused […] Lansky […] speaks for many in asserting that ‘Delusion is restitutive, ameliorating anxieties by altering the construction of reality’. (Roberts [1992], p. 305)

In his own research, Roberts ([1991]) finds that patients with ESDs score higher than patients in remission, rehabilitation nurses, and Anglican ordinands in the ‘purpose in life’ test and the ‘life regard’ index.^2^ The conclusion is that ‘for some there may be satisfaction in psychosis and that [delusion formation] is adaptive’ (Roberts [1991], p. 19). ESDs explain the agent’s puzzling experiences and, depending on their content, they can also play a defensive function, protecting the agent from the acknowledgement of an unpleasant reality or from low self-esteem:

Both the specific contents of delusional beliefs and the experience of having found a powerful and comprehensive explanation, accompanied by a conviction of having discovered the truth, could be preferable to confronting reality again. In these circumstances there would be a movement towards elaboration and chronicity. Thus, discrepancies between delusional and real perspectives are likely to be resolved by further elaboration of delusion and adjustment of life circumstances in order to protect the beliefs from confrontation. A number of theorists with different perspectives have suggested that elaborate delusional systems may, in part, be perpetuated and mediated by the associated psychological benefits. (Roberts [1992], p. 305)

Roberts’s findings are consistent with more recent studies, according to which delusions confer meaning to otherwise deeply puzzling and inexplicable experiences, and help enhance what has been called an overall ‘sense of coherence’.^3^ The sense of coherence is not reduced in people in an acute delusional state (Bergstein *et al.* [2008]). Rather, the sense that one’s life is meaningful might be enhanced with respect to the non-clinical population when the delusional system is elaborated. Sense of coherence and meaningfulness are found to correlate with well-being. In the transition from the acute stage to remission, when the agent’s conviction in the delusion fades and the new explanation for her delusional experiences involves insight into her own psychosis, then the sense of coherence and meaningfulness are reduced, and levels of well-being are also found to drop. When the agent starts doubting the content of her delusion, the realization that she has suffered from a mental illness for years and the world that she lived in was illusory may have negative consequences for her self-understanding and self-esteem (Freeman *et al.* [2004]). Rates of suicide are highest in the first few years of a psychotic illness when people try to come to terms with their fear of chronic mental illness (see Drake and Cotton [1986]; Clarke *et al.* [2006]).

In some cases, agents are able to find additional meaning in life thanks to the formation of a delusion, and their functioning does not seem to be seriously impaired as a result. One such case is Simon, a lawyer with a happy family life and a good career:

[…]out of the blue, he was threatened by a malpractice legal action from a group of his colleagues. Although he claimed to be innocent, mounting a defence would be expensive and hazardous. He responded to this crisis by praying in front of an open bible placed on a small altar that he set up in his front room. After an emotional evening's ‘outpouring’ he found that wax from two large candles on the altar had run down onto the bible marking out various words and phrases (he called these wax marks ‘seals’ or ‘suns’) […] From this time on, Simon received a complex series of ‘revelations’ largely conveyed through the images left in melted candle wax. They meant nothing to anyone else including Simon’s Baptist friends and family. But for Simon they were clearly representations of biblical symbols particularly from the book of Revelations signifying that ‘I am the living son of David … and I'm also a relative of Ishmael and … of Joseph’ […] His special status had the effect of ‘increasing my own inward sense, wisdom, understanding, and endurance’ which would ‘allow me to do whatever is required in terms of bringing whatever message it is that God wants me to bring’. (Jackson and Fulford [1997], pp. 44–5)

Another such case is reported by a clinician in a letter to the *Psychiatric Bulletin*:

Mr A., a 66-year-old man, was admitted following an accidental fall in which he fractured a femur. Following surgery, he expressed bizarre ideas and was referred for a psychiatric opinion. This assessment revealed a long standing complex delusional system in which he believed he was in constant contact with ‘spirits from the other side’. This involved clear auditory hallucinations which occurred frequently and he described the spirits discussing his activities among themselves. He had been having these experiences for over ten years. There was no evidence of persistent mood change nor underlying organic disorder. The illness had begun about five years after his divorce and three years before he retired. He was diagnosed as suffering from late onset or paranoid schizophrenia. Mr A. denied any distressing aspect to his illness and considered himself gifted. He refused to attend for any out-patient follow-up and saw no need for help of any kind. In such cases, which it would seem reasonable to call ‘successful psychotics’, can intervention be justified? (Hosty [1992], p. 373)

Both descriptions of ‘successful psychotics’ stress the role of ESDs in giving the agent a sense of purpose and meaning, and downplay the negative effects on well-being that delusions typically have. This is probably due to the self-enhancing content of the delusions reported (in both cases, people thought of themselves as gifted and invested with special responsibilities) and the support provided by their immediate social circle.

## 5 Learning Resumed

The capacity delusions have to enhance meaningfulness and a sense of coherence are regarded as psychological benefits: they are positively correlated with well-being. But do ESDs have benefits that are not mediated by a positive impact on well-being? One suggestion is that ESDs allow an agent to resume contact with the world after the disruption caused by abnormal experiences. Such benefits have been described in terms of adaptiveness. For instance, ESDs have been described as enabling the agent ‘to remain in *vital* connection with his/her environment’ by Mishara and Corlett ([2009]). This claim is surprising as delusions are often described in the philosophical literature as a departure from reality or a failure in reality testing, but it is justified by reference to three phases in the process by which delusions are formed and consolidated:

1. Anxious expectation: As we saw, in the prodromal stage of psychosis there is an often long period of great anxiety during which the agent is constantly expecting something important to happen. During this period, the processes underlying automated and habitual learning are disrupted due to an incorrect signalling of prediction errors. A prediction error occurs when our experience does not match our predictions: the internal model of the world issuing the prediction is incorrect and needs to be revised. One hypothesis is that, in people with hypersalient experience, prediction-error signals are produced when there is no real mismatch between prediction and actual inputs.
  As a result of excessive prediction-error signals, conscious and controlled processes take over:
  Attention is drawn toward irrelevant stimuli, thoughts, and associative connections which are distressing and unpredictable (McGhie & Chapman [1961]; Kapur [2003]; Uhlhaas & Mishara [2007]). This reflects an impairment in the brain’s predictive learning mechanisms, such that unexpected events, prediction errors, are registered inappropriately (Corlett *et al.* [2007]). (Mishara and Corlett [2009], p. 531)
2. Revelation: Mishara and Corlett ([2009]) argue that when the delusion is formed it puts an end to overwhelming anxiety. The sense of unpredictability caused by the inaccurate coding of a prediction error stops. The stimuli previously experienced as inexplicable and distressing require attention no longer because a suitable explanation has been found for the unpredictable associations. Thus, the processes underlying automated and habitual learning can resume their normal function.
3. Reinforcement of the delusion: To explain how the delusion can be so persistent, the account tells us that the delusion is stamped into the agent’s memory and reinforced every time a new prediction error is registered. The shift back to the habitual and automated learning processes enhances the capacity to respond to cues in the environment and the delusion plays a dominant role in providing explanations for the phenomena previously found to be puzzling and anomalous:
  The delusions […] involve a ‘reorganization’ of the patient’s experience to maintain behavioral interaction with the environment despite the underlying disruption to perceptual binding processes […] At the Aha-moment, the ‘shear pin’ breaks, or as Conrad puts it, the patient is unable to shift ‘reference-frame’ to consider the experience from another perspective. The delusion disables flexible, controlled conscious processing from continuing to monitor the mounting distress of the wanton prediction error during delusional mood and thus deters cascading toxicity. At the same time, automatic habitual responses are preserved, possibly even enhanced. (Mishara and Corlett [2009], p. 531)

More needs to be said about the precise nature of the advantage that the formation of delusions may have, and about whether all or just some ESDs can function in the way proposed by Mishara and Corlett. Notwithstanding the need for further research, the account identifies in some detail the possible effects of delusion formation on perception and cognition, and it is original in offering an argument for the potentially adaptive role of delusion formation. Mishara and Corlett emphasize that the situation in which delusions emerge (hypersalient experience generating anxiety) is already seriously compromised and can develop in even more harmful ways unless the delusion is formed. My next question is whether the formation of delusion, conceived in this way, has any advantages that are distinctly epistemic.

## 6 Epistemic Innocence

In Sections 2 and 3 we saw that delusions in general, and ESDs in particular, are epistemically costly. Here I want to suggest that ESDs can have some epistemic benefits as well as obvious epistemic costs. To consider the potential epistemic benefits of epistemically costly cognitions, I introduce the notion of epistemic innocence. Epistemically innocent cognitions are not necessarily free from epistemic faults, but they do have significant epistemic benefits that would be unattainable otherwise. The notion of innocence I have in mind is analogous to the legal notion of ‘innocence-defence’.

In UK and US law, an innocence-defence is used when an agent is not deemed liable for an act that appears to be wrongful, either because there is no criminal intent (excuse) or because the act does not constitute an offence in the circumstances (justification). Interestingly, justification-defence includes situations in which the act prevents a serious harm from occurring (necessity defence), and it is also referred to as a ‘choice of evil defence’, where the thought is that the agent does not commit an offence because she chooses the lesser of two evils. Here is an example:

Ann swings her arm and injures Ben. She faces moral condemnation and legal liability unless she can offer an explanation that absolves her of full blame […] If Ann acknowledges that she intentionally hit Ben but did so to prevent him from detonating a bomb, she offers a justification. (Greenawalt [1986], p. 89)

One way of fleshing out this notion of innocence is to describe the act as an acceptable response to an emergency situation: Ann’s hitting Ben is an innocent act if, by hitting Ben, Ann prevents him from detonating a bomb, and other ways to stop Ben, such as talking him out of detonating a bomb, are not available to Ann at the time.

My suggestion is that the notion of justification-defence can be used to account for the epistemic evaluation of cognitions that have apparent epistemic costs but also some significant epistemic benefit. When the adoption of a delusional hypothesis helps avoid bad epistemic consequences and adopting another hypothesis would not have the same benefit, then the adoption of the delusional hypothesis is an acceptable response to an emergency situation.

Here are the two conditions for the epistemic innocence of delusions:

Epistemic benefit: The adoption of the delusional hypothesis confers a significant epistemic benefit to a given agent at a given time.
No alternatives: Alternative hypotheses that would confer the same benefit are not available to that agent at that time.

In order to flesh out these conditions, we need to make some assumptions about what counts as an epistemic benefit, and this will depend upon our epistemological commitments. In general, a consequentialist about epistemic evaluation will find the talk of epistemic innocence more congenial than a deontologist, but the notion may be interesting for epistemologists in both camps. For instance, a veritist (that is, a consequentialist who thinks that we should maximize the acquisition and retention of true beliefs) might say that a delusion is epistemically beneficial if it contributes to the acquisition or retention of true beliefs (see Goldman [1986]). A virtue epistemologist (that is, a consequentialist who thinks that we should promote the agent’s intellectual virtues) might say that a delusion is epistemically beneficial if it contributes to the promotion of, say, intellectual curiosity and honesty (see Greco [2012]). A deontologist may be less concerned about the benefits that a delusion brings, as she does not think about epistemic evaluation in terms of the consequences of having a certain cognition (see Booth [2012]); but she may be interested in whether the adoption of other hypotheses was genuinely available to the agent prior to forming the delusion. If the agent’s ability to believe otherwise were compromised, then the deontologist may not regard the agent as responsible or blameworthy for the adoption of the delusional hypothesis.

Different notions and degrees of unavailability can explain the failure to adopt a less epistemically costly hypothesis. This spectrum of possibilities reflects the nature of the limitations that the agent experiences in the relevant context, ranging from standard reasoning limitations affecting all human agents to specific deficits of perception, inference, or memory in clinical settings. The adoption of a less epistemically costly hypothesis may be, strictly speaking, unavailable if the agent cannot even entertain alternative explanations of her experience. In a different scenario, the adoption of a less epistemically costly hypothesis may be available, strictly speaking, but inhibited by motivational factors or biases in belief evaluation. Given that judgements of epistemic innocence apply to a delusion relative to an agent at a given time, and depend on an assessment of the availability of alternatives that is difficult to make in general terms, my aim in Sections 7 and 8 will be to argue that at least some ESDs have the potential for epistemic innocence.

## 7 Epistemic Benefit

I take a broadly consequentialist approach to epistemic evaluation for the purposes of this article, and apply the two conditions to the case of ESDs in the light of the discussion in Sections 4 and 5.

ESDs meet the first condition for epistemic innocence if they bring to the agent a significant epistemic benefit. Here I shall consider whether the beneficial features of ESDs that have been identified in the literature have relevant epistemic consequences. The overall thesis will be that delusions have the potential to meet the epistemic benefit condition if the adoption of the delusional hypothesis supports the agent’s epistemic functionality (that is, her capacity to perform well epistemically), which would be otherwise compromised by the agent’s overwhelming anxiety and her reduced contact with the surrounding environment.

An initial reason to believe that adopting a delusional hypothesis supports epistemic functionality comes from the claim that delusion formation provides some relief from anxiety:

First, endogenous psychosis evolves slowly (not overnight). For many patients it evolves through a series of stages: a stage of heightened awareness and emotionality combined with a sense of anxiety and impasse, a drive to ‘make sense’ of the situation, and then usually relief and a ‘new awareness’ as the delusion crystallizes and hallucinations emerge. (Kapur [2003], p. 15)

As we saw in Section 4, some suggest that unless the inexplicability of salient events characteristic of the delusional mood is resolved, great anxiety and negative emotions can become overwhelming, with adverse effects for well-being. But mounting anxiety and the inability to manage negative emotions would also compromise the capacity to acquire, retain, and use knowledge. One of the chief consequences of anxiety is a lack of concentration, and other consequences—such as irritability, social isolation, and emotional disturbances—negatively affect socialization, making interaction with other people less frequent and less conducive to the productive exchange of relevant information.

One concern with taking anxiety relief to have positive epistemic consequences is that many factors can contribute to anxiety relief over and beyond the adoption of a belief, including a good night’s sleep. Are ESDs on a par with a good night’s sleep in providing an epistemic benefit? They both support epistemic functionality to an extent, but the contribution of a delusion is qualitatively different. If the account of an ESD as a default explanation for anomalous experience is to be trusted, the formation of the delusion puts an end to the uncertainty caused by incorrect prediction-error signalling: delusion formation is a solution to the anxiety caused by hypersalience as opposed to a short-term source of relief, such as a good night’s sleep.

The main problem with considering delusion formation as a solution to anxiety is that although adopting the delusion provides relief from the anxiety due to the hypersalient experience, in the long run the ESD is itself a cause of anxiety due to its often disturbing content and the profound, alienating effects it can have on the agent’s life. Any discussion of the benefits of the adoption of ESDs should not underestimate the psychological and epistemic costs that the maintenance of ESDs has in terms of generating not only anxiety, but also distress, social isolation, and withdrawal. People may no longer feel anxious about their hypersalient experience (for example, ‘how should I interpret this?’), but they can feel anxious and distressed about how the world is according to the delusion (for example, ‘how can I escape from the alien forces persecuting me?’), and suffer the consequences of the social isolation and withdrawal ensuing from reporting the delusion and being met with incredulity (Broome *et al.* [2005]). This is especially true of delusions that are elaborated and systematized as they are believed with great conviction in the acute stage of psychosis and are likely to significantly affect people’s lives. Thus, even after the adoption of the delusion, anxiety and distress may still be a persistent feature of the agent’s experience. Apart from grandiose delusions that are correlated with high self-esteem and low depression (as in the ‘successful psychotics’ who believed that they were especially gifted), other delusions with largely negative content are correlated with high depression and low self-esteem (Smith *et al.* [2006]). This means that the anxiety-relief function of ESDs may be of short duration and, if anxiety relief is the only epistemic benefit an ESD can bring to an agent, then its epistemic innocence will be equally short lived.

Another way in which the adoption of a delusional hypothesis may support epistemic functionality is through engendering a new attitude towards experience. The agent with ESDs no longer finds her experience puzzling, but feels that it is in her power to understand it and that it is important to come to such an understanding. Sense of coherence seems to encompass intellectual curiosity and a sense of self-efficacy and purpose. Arguably, such attitudes are more conducive to the acquisition and exchange of knowledge than the state of passive, anxious uncertainty that characterizes the agent’s experience prior to the formation of the delusion. At the moment this is a speculative claim that needs to be supported by empirical evidence, but it is plausible to suppose that an increased sense of coherence enables agents to view their own experiences as interesting and worth investigating, leading to a more active engagement with the physical and social environment and, potentially, to the acquisition of new true beliefs.

A third contribution that the adoption of a delusional hypothesis can make to epistemic functionality derives from the argument that ESDs may enable automated learning to resume after the disruption caused by incorrect prediction-error signalling. As we saw in Section 5, Mishara and Corlett ask whether delusion formation in the context of schizophrenia can preserve and even enhance learning. They argue that the attention and control dedicated to the unpredictable hypersalient events detract from the capacity an agent has to learn and remember. The uncertainty caused by the unexpected associations causes conscious and controlled processes responsible for learning to focus on the stimuli that seem perplexing or threatening at the expense of the other stimuli that end up being neglected. When the delusion is formed, it functions to release attention, and causes habitual and automated processing to resume. This suggests that the formation of the delusion ‘frees up’ the agent’s cognitive resources, which can be then deployed to successfully make sense of the rest of the surrounding environment.

ESDs promoting a new attitude towards experience and enabling learning to resume come with considerable epistemic costs that should not be underestimated. Following the formation of a delusion, the agent interprets all unexpected and salient events in the light of the delusion, and counter-arguments do nothing but reinforce the belief that the content of the ESD is true. As we noticed, the agent may have a renewed willingness to investigate and greater cognitive resources to carry out such an investigation, but she does not approach the world with an open mind. The agent’s experience is likely to be interpreted via the same delusional hypotheses that have crystallized into persistent delusional beliefs. Every time a new salient fact is confronted, ‘there is a “monotonous” spreading of the delusion to new experience’ (Mishara and Corlett [2009], p. 531). Thus, the newly acquired information is unlikely to give rise to knowledge if the ESD plays the role of the dominant explanatory framework.

The analysis above shows that some of the features of ESDs have positive epistemic consequences, in terms of the agent benefiting from better allocation of cognitive resources and increased concentration, socialization, and willingness to investigate, than in the prodromal stage of psychosis. However, the formation of delusions also carries significant epistemic costs that are unlikely to be outweighed by the benefits I described. Moreover, the potential epistemic benefits I discussed here depend on the agent being in an already seriously compromised epistemic state prior to the delusion being formed. The adoption of the delusional hypothesis may be beneficial because it prevents the occurrence of a disastrous epistemic breakdown at a time when experience is anomalous, hypersalience causes anxiety, and the disruption of prediction-error signalling leads to compromised automated learning.

It is not surprising that the epistemic benefits of delusions do not outweigh their costs. Delusions are not epistemically good after all. But they may still count as epistemically innocent if they deliver a significant epistemic benefit that could not be attained otherwise. This would occur because, in the conditions generated by the hypersalience of unpredictable stimuli, non-delusional hypotheses are in some sense unavailable to the agent as candidate explanations. Arguably, not adopting any hypothesis that explains the agent’s experience, relieves anxiety, increases meaningfulness, and allows learning to resume would be (epistemically) worse than adopting the delusional hypothesis. If the agent did not form the delusion, she would be locked in a perpetual delusional mood characterized by hypersalience. Delusions would count as the only way for the agent to partially restore her already compromised epistemic functionality.

## 8 No Alternatives

In order to offer some plausibility to the claim that ESDs have the potential for epistemic innocence, I have presented them as explanations of puzzling experiences. But could non-delusional explanations make sense of the experience and confer the relevant epistemic benefits without incurring the numerous epistemic costs that delusions have? It is likely that non-delusional explanations of hypersalient experiences in the prodromal stage of psychosis would be less bizarre, and less at odds with the agent’s other beliefs than the delusional explanation. However, they would also be less likely to make sense of the puzzling nature of the hypersalient experience in a way that relieves anxiety, increases the sense of meaningfulness and purpose, and provides a default account of prediction errors.

How to characterize the availability of the non-delusional hypotheses to agents in the prodromal stage of psychosis is an issue that deserves greater attention than I can give it here, but in general terms one may refrain from adopting a hypothesis if information supporting it is opaque to introspection, if the hypothesis has negative motivational charge, or if it not open to consideration or evaluation due to reasoning biases or deficits. If alternative explanations are not available to the agent adopting a delusion, then the case for the epistemic innocence of ESDs would be stronger, as ESDs would easily meet condition two. At that point, it would be plausible to suggest that having no explanation for deeply distressing and puzzling experiences would be worse than having a delusional one (recall my previous quote from Jaspers: ‘No dread is worse than that of danger unknown’). One powerful benefit of adopting the delusion would be to have one hypothesis explaining hypersalient experiences as opposed to none.

While the unavailability of alternative hypotheses after the adoption of the delusional explanation largely depends on the way the agent’s experience and reasoning are affected by the delusion itself,^4^ the unavailability of alternative hypotheses prior to the adoption of the delusional explanation cannot depend on the agent being delusional. Several accounts of delusion formation lend *prima facie* support to the view that delusional hypotheses at that stage are somehow inescapable. In the literature defending the two-factor theory (Davies *et al.* [2001], p. 153; Aimola Davies and Davies [2009], p. 291), delusions are characterized as prepotent doxastic responses to the agent’s perceptual experience. This may be either because the content of the delusion is already fully encoded in the content of the experience and the delusion is just a default endorsement of the experience as veridical (‘seeing is believing’, as the authors put it); or because there is a difference in specificity between the content of the experience and the content of the delusion, but the delusion appears to the person to be the best explanation of the experience and thus it is accepted, leaving no room for alternative explanations (Coltheart *et al.* [2010]).

Additional evidence that alternative (non-delusional) hypotheses explaining the experience may not be available comes from a study by Freeman and colleagues suggesting that people in the acute stage of psychosis may be blind to alternative hypotheses:

Three quarters of the patients reported that there was no alternative explanation for their experiences. The delusion was their only explanation. This matches with clinical experience. Nevertheless, it is a striking finding. By definition a delusional belief is highly improbable. The evidence cited for a delusion is, at best, ambiguous. Yet most individuals could not report any potential alternative explanation for the ambiguous evidence however unlikely that they considered the alternative. (Freeman *et al.* [2004], p. 677)

Freeman and colleagues suggest that the unavailability of alternative hypotheses may be due to a variety of factors. First, if we are thinking about the adoption of the delusional hypothesis, then the nature of the delusional experience often seems to provide information about the external world as opposed to reflecting something that is happening to the agent herself (for example, hearing voices), and thus internal explanations—such as, ‘there must be something wrong with me’—do not appear as good candidates.

Second, reasoning biases such as jumping to conclusions and the need for closure make it much easier for people to accept the first suitable hypothesis that comes to mind, without waiting for further evidence (Broome *et al.* [2007]). Although such biases are more frequently observed in people at high risk of developing psychosis, they are not themselves a sign of mental illness and are common in the non-clinical population as well.

Finally, one powerful consideration is that motivational factors are likely to interfere with the acceptance of alternative hypotheses, especially when the content of the delusions is not too distressing (as in ‘successful psychotics’). Depending on the content of the delusion and its impact on the agent’s well-being, there may be few incentives for the agent to believe something that implies that she must be mad (Roberts [2006]; Freeman *et al.* [2004]).

The three forms of unavailability I considered here (that is, unavailability due to bad explanatory fit, unavailability due to reasoning biases, and unavailability due to motivational reasons) do not seem to depend on mental illness, or the presence of psychotic symptoms. First, the tendency to prefer hypotheses that explain the details of one’s experience in a more satisfactory way is not confined to the clinical population. Some of the experience people with delusions need to account for is anomalous, but having anomalous experiences is neither sufficient nor necessary for mental illness in general, nor for psychosis in particular. Second, although reasoning biases such as jumping to conclusions seem to be more accentuated in a clinical sample of people likely to report delusions, they are neither unique to that sample, nor markers of mental illness. Finally, any agent, with or without psychosis, would prefer not to adopt a hypothesis that has negative implications for her self-concept, and the hypothesis that she is mentally ill is obviously unpleasant and disruptive.

What we know about the adoption of delusional hypotheses suggests that it is certainly difficult for alternative hypotheses to exhibit the same explanatory power and play the same anxiety-relief function as the delusional hypothesis. Alternative hypotheses to the delusional one that meet such desiderata may not carry fewer epistemic costs than those associated with adopting the delusional hypothesis. Thus, it is likely that some ESDs will meet condition two for epistemic innocence, even if this has not been conclusively shown. The hope is that raising the possibility that delusions have epistemic benefits that are otherwise unattainable will inspire further empirical work and will enable us to arrive at a more definite answer.

## 9 Conclusions and Implications

In this article I considered whether ESDs have the potential for epistemic innocence, where epistemic innocence is characterized as the epistemic status of those cognitions that, despite their epistemic costs, have significant epistemic benefits that would be otherwise unattainable. I argued that there are good prospects for those ESDs that allow the agent to escape from a paralysing state of hypersalience and that provide relief from anxiety, enhance meaningfulness, and enable automated learning to resume.

A man sees a dog raising its paw in front of a church and comes to believe that God has sent him a message via the dog.^5^ Arguably, the belief that God wants to communicate with him makes the man feel valued and important, and provides a potentially unifying explanation for a number of apparently random events previously experienced by him as salient. We can speculate that the desire to understand God’s message will lead the man to pay closer attention to his surroundings, although this may result in the formation of other implausible (possibly delusional) beliefs. We can also speculate that, without any explanation of the dog’s movement, the sense that the event is salient and important but has no obvious interpretation may lead to anxiety and self-doubt.

When we think about inaccurate cognitions that may have psychological benefits—such as self-deception, delusional beliefs, positive illusions, confabulatory narratives, and distorted memories—it is tempting to think in terms of a trade-off. Believing something false or putting a positive spin on a past event can make us feel better about ourselves, but it leads us further away from the truth. Thus, it may be pragmatically advantageous, even adaptive, but is not epistemically good. Do we really get pragmatic benefits at the expense of epistemic ones? Do people with delusions gain anxiety relief or an enhanced sense of meaning at the cost of foregoing the truth, that is, the real explanation of their own experiences?

My discussion suggests that it is too simplistic to endorse the trade-off view in the case of ESDs, because some of the psychological benefits and adaptive features attributed to delusions can carry significant epistemic benefits that it would be unwise to neglect. Thus, a general lesson for epistemology from the delusions literature seems to be that epistemically costly cognitions should not be dismissed as bad without further consideration, and should not be challenged by default. Rather, we should pay attention to the potential epistemic function such cognitions may have and acknowledge that, in some contexts, they may deliver epistemic benefits as well as costs. Their positive function does not necessarily translate into their being epistemically good or justified, as their costs may still outweigh their benefits. But if the benefits are significant, and difficult or impossible to obtain by other means, then such cognitions may gain epistemic innocence and may be tolerated while they play their positive function. And if delusions have positive as well as negative epistemic features, this should be reflected in the way they are defined and characterized. As we saw in Sections 2 and 3, delusions are largely defined on the basis of their negative epistemic features, in the psychological literature as well as in diagnostic manuals. An understanding of their positive epistemic features will change the way we define delusions and distinguish them from other cognitions and other symptoms of psychiatric disorders.

The possibility that delusional beliefs play a positive epistemic function for some agents in some contexts will help determine whether it is a good strategy to challenge such beliefs. It has long been recognized that challenging delusions is not always clinically useful:

Challenging or evaluating delusional explanations should be done only in the context of an alternative explanation that the patient finds acceptable. Our preference is for building up an alternative explanation to the delusion that is not depressogenic and that is based on a biopsychosocial framework, and for using confirmatory reasoning to strengthen the degree of endorsement. (Freeman *et al.* [2004], p. 679)

If there is no alternative hypothesis the agent finds acceptable, then challenging the delusion does not help clinically. Freeman and colleagues argue that it is better to spend time and energy making available to the agent an alternative hypothesis that shares some of the psychological benefits of delusions. Would this strategy also be in the epistemic interests of the agent? So it seems, given what we saw in Sections 7 and 8. Not having any explanation available for salient and puzzling experiences may impair the agent’s epistemic functionality to a greater extent than endorsing a delusional explanation.

Isn’t the appeal to the epistemic benefits of ESDs superfluous in a clinical perspective? If, at a given time, challenging a delusion is not the best option for the agent’s well-being, it would seem not to matter whether the delusion also has epistemic benefits at that time. But think about a clinical team making the decision not to challenge a delusion to avoid the risk of the agent becoming depressed from insight into her mental illness.^6^ We may feel that by not challenging a delusion, or not revealing to the agent what we think is the true explanation of her puzzling experience, we are placing the agent at an epistemic disadvantage. In line with the trade-off view I mentioned earlier, the agent’s well-being is being preserved at the expense of her access to the truth. The description of the case changes, however, if the delusion meets the conditions for epistemic innocence: the trade-off view no longer captures the complexity of the situation. What is psychologically beneficial may also be (to some extent and in the short term) epistemically beneficial. It may be ill-advised to challenge the delusion if, for that agent at that time, the delusional hypothesis serves a useful epistemic function, allowing the agent who has endorsed it to navigate the world, albeit in an imperfect way. In other words, not only does the proposed notion of epistemic innocence create the conceptual space to acknowledge the epistemic benefits of delusions, enriching the vocabulary of epistemic evaluation, but its application can also inform our epistemic practices and contribute to the process through which we decide when and how to challenge delusional beliefs.

## Footnotes

^1^ It is the default position in psychology and psychiatry to regard delusions as beliefs. In philosophy there is a lively debate between doxasticists (who regard delusions as beliefs) and anti-doxasticists (who regard delusions as other than beliefs). In this article I assume that it is plausible to regard delusions as beliefs. A proper defence of the doxastic nature of delusions cannot be offered here (see Bortolotti [2010]; Bayne and Pacherie [2005]), but this does not compromise the general appeal of the main thesis of the article. In the most popular anti-doxastic accounts of delusions it is acknowledged that delusions involve beliefs (among other cognitive or affective states) or that delusions are sufficiently belief-like to be subject to epistemic evaluation (see Currie and Jureidini [2001]; Schwitzgebel [2012]). That is why the view that delusions have epistemic benefits is not incompatible with anti-doxasticism about delusions.

^2^ The purpose in life test, as the name suggests, measures a person’s experience of meaning and purpose in life (Seeman [1991]). It is a twenty-item scale and each item is rated on a seven-point scale. Total scores range from 20 (low purpose) to 140 (high purpose). Here are some items: ‘I am usually: completely bored (1) — exuberant, enthusiastic (7)’; ‘If I could choose, I would: prefer never to have been born (1) — like nine more lives just like this one (7)’; ‘As I view the world in relation to my life, the world: completely confuses me (1) — fits meaningfully with my life (7)’. The life regard index, again as the name suggests, measures a person’s regard for her life (Battista and Almond [1973]). It is made of twenty-eight items divided into two subscales: the first measures the ability of the person to see her life within some framework, and to have derived a set of life goals or a purpose in life from them; the second measures the degree to which the person sees herself as having fulfilled, or being in the process of fulfilling her life goals. People rate statements based upon their feelings on a five-point scale ranging from 1 (agree) to 5 (disagree). Sample statements are: ‘I have a very clear idea of what I’d like to do with my life’ and ‘I don’t really like what I’m doing’. The purpose in life test and the life regard index are both widely used and regarded as reliable means for measuring important aspects of the sense of meaning and purpose in people’s lives.

^3^ The sense of coherence is defined as ‘a global orientation that expresses the extent to which one has a pervasive, enduring though dynamic, feeling of confidence that (1) the stimuli deriving from one's internal and external environments are structured, predictable, and explicable; (2) the resources are available to one to meet the demands posed by these stimuli; and (3) these demands are challenges, worthy of investment and engagement’ (Antonovsky [1987], p. 91).

^4^ Klaus Conrad argues that ‘no alternative explanatory frame’ is available to the person with schizophrenia who has had the ‘revelation’ and now endorses a delusional explanation of her experience. Conrad’s view is described as follows: ‘At the aha-moment, the patient is *unable* to shift “frame of reference” to consider the experience from any other perspective than the current one. The transition from delusional mood to the Aha-Erlebnis of the delusional revelation occurs precisely at the moment of *loss* of the patient’s ability to distance from the experience’ (Mishara [2010], p. 10, emphasis added). In some versions of the two-factor theory of delusions, it is argued that people with delusions fail to evaluate already adopted beliefs in light of new evidence (Coltheart *et al.* [2010]). This supports the thought that, once formed, the delusion is difficult to reject.

^5^ This example is adapted from a real-life case (Leeser and O’Donohue [1999]).

^6^ The case of reverse Othello syndrome (Butler [2000]) is a good example of this.
